# Does an immigrant teacher help immigrant students cope with negative stereotypes? Preservice teachers' and school students' perceptions of teacher bias and motivational support, as well as stereotype threat effects on immigrant students' learning

**DOI:** 10.1007/s11218-023-09793-z

**Published:** 2023-06-10

**Authors:** Madita Frühauf, Johanna Hildebrandt, Theresa Mros, Lysann Zander, Nele McElvany, Bettina Hannover

**Affiliations:** 1 Department of Education and Psychology, Freie Universität Berlin, Berlin, Germany; 2 Leibniz Universität Hannover, Hannover, Germany; 3 Institut Für Schulentwicklungsforschung IFS (Institute for School Development Research), Technische Universität Dortmund, Dortmund, Germany

**Keywords:** Ethnic minority teacher, Teacher bias, Teacher support, Stereotype threat, Turkish origin students, Immigrant students

## Abstract

Can immigrant school students profit from an immigrant teacher sharing their minority background? We investigate preservice teachers' (Study 1; *M*_*age*_ = 26.29 years; 75.2% female) and school students' (Study 2; *M*_*age*_ = 14.88 years; 49.9% female) perceptions of a teacher as well as immigrant school students' learning gains (Study 2) by comparing four experimental video conditions in which a female teacher with a Turkish or German name instructs school students in a task while either saying that learning gains differed (stereotype activation) or did not differ (no stereotype activation) between immigrant and non-immigrant students. Study 1 shows that preservice teachers, regardless of their own cultural background, perceived the Turkish origin teacher as less biased, even when she voiced the stereotype, and as more motivationally supportive of school students in general than the German origin teacher. Study 2 shows that in contrast, among school students, the minority teacher was not perceived as less biased than the majority teacher. Rather, immigrant school students, in particular those with Turkish roots, were more concerned than students of the German majority that the teacher—irrespective of her background—was biased. Interestingly, these differences between students from different backgrounds disappeared when the teacher said that learning gains differed between immigrant and non-immigrant students. Immigrant school students of non-Turkish backgrounds, but not Turkish origin students suffered in their learning when instructed by the Turkish origin teacher who voiced the stereotype. We discuss implications for teacher recruitment.

## Introduction

Educational systems around the world struggle how best to support immigrant students while reducing ethnic inequalities (Stanat & Christensen, 2006). Compared to their non-immigrant peers, on average immigrant students reach lower levels of educational participation and academic competencies, are overrepresented at lower track schools, and gain lower quality school leaving certificates (European Commission, 2019; for Germany, see Weis et al., 2019). In Germany, where our study was conducted, students of Turkish descent are of particular concern as they represent the largest subgroup of immigrant students (Statistisches Bundesamt, 2021) and as for them the achievement gap is particularly severe, even after controlling for social and educational background characteristics (e.g., Aktionsrat Bildung, 2016; Autorengruppe Bildungsberichterstattung, 2016; Kristen et al., 2019). One of the many empirically substantiated causes why immigrant students in general and students of Turkish origin in particular underperform in the German school system (cf. Kristen et al., 2019) are identity-threats emanating from negative stereotypes (Steele, 1997) that impair learning and achievement when activated in the context of performance (cf. Appel et al., 2015).

In education policy, it is often argued that immigrant students would profit from greater representation of teachers from ethnic minority backgrounds (for Germany see, e.g., Senatsverwaltung für Bildung, Jugend und Familie (2020); Georgi et al., 2011; for other countries, see Morgenroth et al., 2015). Back in 2008, when Barack Obama became the first Black president of the United States, politicians, educators, parents, and students hoped that he would be a role model supportive of African Americans (Marx et al., 2012). As Morgenroth et al. (2015) write, such “role models are often regarded as a panacea for inequality, by the general public, policymakers, and the academic literature alike” (p. 465).

In a recently published study from Germany, however, no performance advantages were found for immigrant students being taught by an immigrant teacher: Neugebauer et al. (2022) compared the test scores in reading, listening, and orthography in the German language in 9262 ninth graders either instructed by a teacher with an immigrant background (*n* = 62) or a teacher with no immigrant background (*n* = 422). For none of the observed outcomes did the authors detect an advantage for immigrant students instructed by an immigrant teacher. Neugebauer et al. (2022) even found that reading test scores were significantly lower for immigrant students when they were taught by an immigrant teacher than when their teacher had no migration background. Analyzing a large sample of 5-year-olds in Germany, Neugebauer and Klein (2016) did not find any performance differences in immigrant children's competencies in the German language, in mathematics, or science depending on whether they were taught by an immigrant or a non-immigrant teacher.

The fact that these studies—and no other study of which we are aware—did not demonstrate any improvements in the achievement of immigrant students as a result of being taught by an immigrant teacher need not invalidate the assumption that minority teachers have a beneficial impact on the academic development of minority students. There are numerous intermediate steps between teacher characteristics, such as their immigrant background on the one hand and student performance outcomes on the other, that have not yet been fully identified in research. Possibly, while not directly supporting immigrant students' achievements, teachers with an immigrant background more likely provide identity-safety—for students of all backgrounds. Cohn-Vargas and Steele (2016) coined the term identity-safety as “an antidote to stereotype threat” (p. 25). In an identity-safe classroom, each student—irrespective of their background—assumes that the teacher is unbiased toward them, has high expectations, and wants to support them in their learning to the best of their ability, while embracing cultural, linguistic, and skill diversity as a resource for learning (Steele & Cohn-Vargas, 2013). A teacher providing identity-safety is aware of and sensitive to the differences among students according to their diverse backgrounds and, in order to motivate every single student, adapts his or her teaching practices accordingly. Thus, the question arises whether students associate different levels of capability to teachers with and without an immigrant background in creating an identity-safe space.

### The impact of stereotypes on immigrant students in classrooms that are not identity-safe

Students with a minority background are less likely to experience identity-safety at school than other students. Several studies reported negative stereotypes about immigrants in general and about people of Turkish descent living in Germany in particular (cf. Appel et al., 2015). Stereotypes are defined as generalized beliefs about characteristics and behaviors of groups and can influence and affect individualized information processing in interpersonal encounters (Fiske & Neuberg, 1990). In a study systematically comparing stereotypes toward different groups of immigrants in Germany, Froehlich and Schulte (2019) found that university students thought that most Germans would perceive Turkish immigrants as much less competent than Germans and as less competent than other immigrant groups, such as Italians, Russians, and Poles. A study conducted about ten years earlier showed a similar pattern: German university students perceived Turkish immigrants as significantly less competent than Germans (Asbrock, 2010). König et al. (2022) had adolescents from Germany workin on an implicit association test and found that, on average, German majority adolescents and adolescents with a non-Turkish immigrant background held negative attitudes toward people with Turkish roots. Stang et al. (2021) found that, already in fourth grade, German majority children had negative implicit attitudes toward people with a Turkish immigrant background.

Accordingly, as one potential factor contributing to the underachievement of Turkish immigrant students in Germany, stereotype threat—the opposite of identity-safety—has been discussed (Appel et al., 2015; Baysu & Phalet, 2014; Froehlich et al., 2018; Martiny et al., 2014). Stereotype threat describes the phenomenon that when a negative performance-related stereotype is activated in a learning or achievement context, individuals to which the negative stereotype pertains underperform (Steele, 1997), due to the impairment of working memory efficiency (Schmader et al., 2008). As substantiated by reviews and meta-analytic findings, stereotype threat contributes to ethnic minority or immigrant students' educational disadvantage (Appel et al., 2015; VanLandingham et al., 2022; Weber et al., 2018). Martiny et al. (2014) found that when being told that German and Turkish origin students differed in their mathematics ability, students with Turkish roots attending school in Germany underperformed in a mathematics test. Froehlich et al. (2016) reported that students of Turkish descent in Germany underperformed in a verbal ability test when it was described as diagnostic of verbal intelligence, to the extent that students saw intelligence as fixed. These experimental studies suggest that there is “a threat in the air” (Steele, 1997, p. 13) for Turkish origin students going to school in Germany—which we will try to examine in more detail in the present research.

### Bias toward immigrant students in ethnic majority and minority teachers

According to the social identity approach (Tajfel & Turner, 1979), in intergroup encounters people discriminate between ingroup and outgroup in their pursuit of positive distinctiveness and are positively biased toward ingroup members and negatively biased toward outgroup members in their perception and treatment. Favoring ingroup members and discriminating against outgroup members can bolster individuals' self-esteem in intergroup comparisons (Tajfel & Turner, 1979). The social identity approach can explain why ethnic majority teachers in Germany have been found to be negatively biased (e.g., Costa et al., 2022; Lorenz et al., 2016) or less positively biased (Kaiser et al., 2017; Tobisch & Dresel, 2017) toward ethnic minority students than toward ethnic majority students. For instance, Lorenz et al. (2016) found that, right after the start of the school year, teachers' expectations for academic attainments in the school subject German were negatively prejudiced against Turkish origin students. Similarly, Lorenz (2021) demonstrated that teachers had lower expectations in the linguistic domain toward Turkish origin students than would have been predicted by students’ reading skills, cognitive abilities, motivation, socioeconomic background, and gender. Further, to the extent that teachers agreed with negative achievement-related stereotypes about Turkish origin students, they also expected lower linguistic achievements for individual Turkish students.

The social identity approach can also explain why ethnic minority teachers, as compared to teachers of the German majority, have been found to be less negatively biased or even positively biased toward immigrant students. For instance, in a study by Gegenfurtner (2022) preservice teachers with an immigration background reported more positive attitudes toward immigrant students than German ethnic majority teachers. Similarly, Glock and Kleen (2019) found that ethnic minority preservice teachers had more positive implicit attitudes toward Turkish origin students than German origin preservice teachers. Glock and Schuchart (2020) observed that preservice teachers of Turkish origin judged a student described by a Turkish first name as more proficient in Turkish and more popular with his peers than did German origin preservice teachers and preservice teachers from diverse origins. Also, preservice teachers of Turkish origin were less inclined than preservice teachers of German or diverse origins to associate mathematic ability, science ability, and competence in general more strongly with ethnic majority students than with students with foreign roots. Kleen et al. (2019) found that, while preservice teachers' ethnicity did not affect explicit attitudes toward Turkish origin students, an implicit measure of attitudes toward Turkish origin students (which is robust to social desirability concerns) revealed more negative attitudes among German origin teachers compared to teachers of various ethnic minority backgrounds, who in turn had the most positive attitudes toward students of their ethnic ingroup.

### Teacher motivational support for immigrant and non-immigrant students

Research conducted in German schools also suggests that minority teachers can be particularly motivating, not only for immigrant students but for students of all backgrounds. Especially immigrant students can be expected to feel motivated and engaged by a teacher who enjoys teaching immigrant students, with (preservice or trainee) teachers with an immigrant background reporting more positive attitudes, more positive emotions, higher self-efficacy toward teaching ethnically diverse school classes (Hachfeld et al., 2012; Syring et al., 2019) and a stronger endorsement of cultural diversity (Hachfeld et al., 2012) than German ethnic majority teachers.

Immigrant and non-immigrant students alike can be expected to profit from a teacher who holds multicultural beliefs, i.e., who considers cultural diversity as a resource when designing lessons and is convinced that s/he can provide equal opportunities for ethnic minority and majority students by being sensitive to and appreciating the differences between them (Hachfeld et al., 2012, 2015), rather than ignoring students' backgrounds (so called egalitarianism, Civitillo et al., 2021): For German schools, Schachner (2019) reported that cultural pluralism, i.e., embracement of students’ diverse cultural backgrounds as a resource, as perceived by the students, was positively associated with student adaptation outcomes (e.g., integrated sense of identity, positive interethnic relations) for both, ethnic minority and ethnic majority students. Similarly, Schwarzenthal et al. (2018) found that different types of cultural diversity norms at school in Germany—i.e., equality norms (which emphasize contact and cooperation between groups and the rejection of prejudice and racism) and cultural pluralism norms (which emphasize the value of diversity) as perceived by the students—were generally associated with more positive adaptation outcomes in students with and without immigrant background.

### Minority teachers prevent negative effects of stereotypes on stigmatized students

Minority teachers can set off or counteract the threat posed by negative stereotypes. Chaney et al. (2018) argue that negative stereotypes about one's ingroup and low representation of ingroup expert role models is an accumulation of identity-threats for stigmatized individuals which signalize them that they do not belong. In this situation, an ingroup expert role model—such as a teacher—is a cue for stigmatized learners that the probability of being negatively stereotyped is low as that expert is expected not to endorse the stereotype about the shared ingroup. As ingroup expert role models, minority teachers create identity-safe environments in which stigmatized learners' motivation is not hampered by identity-threats: “Identity-safe environments challenge the validity, relevance, or acceptance of negative stereotypes linked to stigmatized social identities” (Davies et al., 2005, p. 278). Minority teachers demonstrate through their personal educational trajectories “that their stigmatized social identities are not a barrier to success in targeted domains” (Davies et al., 2005, p. 278). Consistent with this view, Liu et al. (2021) identified the provision of ingroup expert role models as an effective strategy to prevent stereotype threat. The expert role model makes stigmatized individuals aware of similar and successful others who disconfirm the negative stereotype of their own group. Liu et al. (2021) found moderately strong performance improvements (*d* = 0.63) among stigmatized individuals across 26 experimental interventions that used ingroup role models to support learners when they were exposed to stereotype threat.

## The present research

### Teacher bias and teacher motivational support

As reported above, previous research from Germany suggests that immigrant teachers are supportive of students in general and of immigrant students in particular because they are less negatively biased toward immigrant students (Gegenfurtner, 2022; Glock & Kleen, 2019; Glock & Schuchart, 2020; Hachfeld et al., 2012; Kleen et al., 2019), more motivated to teach ethnically diverse school classes (Hachfeld et al., 2012; Syring et al., 2019), and endorse multicultural beliefs to a stronger extent (Hachfeld et al., 2012) than German ethnic majority teachers. However, studies investigating student outcomes did not find performance advantages when immigrant students were instructed by an immigrant (rather than a non-immigrant) teacher (Neugebauer & Klein, 2016; Neugebauer et al., 2022). One explanation is that perhaps, students need to explicitly know their teacher has an immigrant background to profit from the identity-safety s/he can provide. It is also possible that the influence of other teacher characteristics—e.g., socioeconomic status (c.f. Ostermann & Neugebauer, 2021) or sense of belonging (Wolf et al., 2021)—are confounded with the influence of the teacher's migration background (a possible explanation for the negative effects on student outcomes found for teachers with an immigrant background by Neugebauer et al., 2022). In our own research we used an experimental manipulation to tell students that the teacher in a video tutorial who instructed them in a task either did or did not have an immigrant background. In this way, we ensured that students were aware of the teacher's background and at the same time we were able to keep other possibly confounded characteristics of the teacher constant.

Also, while, as reported above, in several studies the attitudes immigrant and non-immigrant (preservice) teachers in Germany hold towards immigrant students were directly compared (Gegenfurtner, 2022; Glock & Kleen, 2019; Glock & Schuchart, 2020; Hachfeld et al., 2012; Kleen et al., 2019; Syring et al., 2019), there is no research on the attitudes which (preservice) teachers have about immigrant and non-immigrant teachers: we know nothing about whether the people concerned themselves, namely (prospective) teachers, also believe that teachers with an immigrant background can support students in a special way. As the call for more teachers with an immigrant background has been repeatedly voiced in politics in recent years (cf., Berlin network for teachers with migration background Berlin.de), it can be assumed that (preservice) teachers—irrespective of whether they themselves have an immigrant background or not—also perceive immigrant teachers as particularly helpful for students. Following social identity theory (Tajfel & Turner, 1979), however, it is also conceivable that teachers favor their own origin group: i.e., German origin teachers have a more positive perception of German origin teachers and teachers with an immigrant background of immigrant teachers.

To investigate whether a teacher with a minority background is perceived as particularly likely providing identity-safety to students in an experimental design, we had preservice teachers (Study 1) and school students (Study 2) with and without an immigrant background watching a video^1^ either showing a minority Turkish origin teacher or a majority German origin teacher instructing school students in a vocabulary learning task. Participants were asked to what extent they thought of the teacher as (a) unbiased and (b) motivating for students.

### Protection against stereotype threat by the teacher

As reported above, many studies found (e.g., Martiny et al., 2014; for meta-analyses, see Appel et al., 2015; Nguyen & Ryan, 2008) that a mere reference to differences in minority and majority students' achievements is experienced as stereotype threat by minority students even when the direction of the difference is not explicitly mentioned (moderately explicit stereotype activation; Nguyen & Ryan, 2008). As also reported above, minority teachers invalidate negative stereotypes and therefore buffer negative effects of the activation of stereotypes on stigmatized students' performance (Chaney et al., 2018; Liu et al., 2021). To our knowledge, none of the many studies on stereotype threat differentiated effects according to whether the difference in achievements between minority and majority group was mentioned by a member of the stigmatized minority group or a member of the majority group. It is possible that stereotype threat effects are mitigated or even absent when the person voicing the stereotype is a member of the stigmatized group and thus signals identity-safety.

To test this assumption, in our videos^1^ we combined the experimental manipulation of teacher origin (Turkish vs. German) with a manipulation of stereotype threat, comparing preservice teachers' (Study 1) and school students' (Study 2) perception of teacher bias and teacher motivational support across the four experimental conditions. In Study 2, we additionally investigated whether stereotype activation differentially impacts school students' learning gains depending on the teacher's background.^2^

## Study 1

### Research hypotheses

The following research hypotheses were investigated in Study 1.

#### Hypothesis 1

Preservice teachers perceive the Turkish origin teacher to be (*H1a*) less biased toward immigrant students, and (*H1b*) more motivating for all students than the majority German origin teacher.

#### Hypothesis 2

Preservice teachers perceive the German origin teacher as more biased (*H2a*) and less motivating (*H2b*) if she addresses the stereotype (compared to when she does not activate the stereotype), while the perception of the Turkish origin teacher is unaffected by her addressing or not addressing the stereotype.

We further explored, with no directional hypotheses, whether preservice teachers with German, Turkish, or other non-German family language would differ in their perception of the teacher shown in the videos.

### Methods

#### Research participants

A total of 505 preservice teachers enrolled in a teacher education master program at Freie Universität Berlin took part. Four hundred and sixty-eight participants gave their consent for the scientific use of their data of which 311 (66.5%) indicated German, 50 (10.6%) Turkish, and 107 (22.9%) a language other than German or Turkish to be their family language (see below for operationalization of family language). Three hundred and fifty-two participants identified as female (75.2%), 112 as male (31.8%) and 4 participants indicated the gender diverse. This distribution represents the distribution of the genders among teachers at general education schools in Germany well (Statistisches Bundesamt, 2021).^3^ Of the respondents, 26.1% indicated that they were already working part-time at a school, in parallel to their university studies. When calculating participants' mean age, we excluded unrealistic data (below 20 and above 60 years of age, *n* = 15). The mean age of the participants was 26.29 years (*SD* = 6.28).

#### Experimental design and procedure

Data was collected using an online questionnaire. Participation was advertised as part of a large lecture. As an incentive for participation, students were promised a report on the results. The questionnaire was scheduled to take 25–30 minutes to complete. In a brief written introduction to the study, it was said that the study was about what preservice teachers think about how a teacher is perceived by their students. The participant was asked to judge a teacher from school students' perspective who would be shown in a video. Next, a video was presented (cf. Ollrogge et al., 2022) showing a teacher acting in front of a blackboard in a typical classroom setting. The teacher explained a learning task on German vocabulary to the viewer (for details see Study 2). As the majority of teachers in Germany are female^3^, the teacher was played by a female actress.

##### Manipulation of teacher origin

The actress that played the teacher in the video was a young German woman who, by appearance, could be perceived as of Turkish or of German descent. Right at the beginning of the video the teacher presented herself to the viewer either with a typical Turkish name and background (“My name is Merve Yıldırım. I’m a teacher and live in Berlin. My parents are from Turkey. Before I was born, they moved to Berlin and we live here ever since.”) or with a typical German name and background (“My name is Julia Schmidt. I’m a teacher and live in Berlin. My parents are from North Rhine-Westphalia. Before I was born, they moved to Berlin and we live here ever since.”). In order to increase the salience of the teacher's origin, she wrote her name on the blackboard in the fictitious classroom.

##### Manipulation of stereotype activation

In the video, the manipulation of teacher origin was followed by the stereotype activation manipulation. In the stereotype activation condition, the teacher said: “In the word learning task, you are shown difficult German words. In the past, research has been done on how successful young people are in this learning task. This has shown the following: The learning success of adolescents who speak only German at home *differed* from the learning success of adolescents who also speak Turkish at home.” The teacher did not indicate the direction of the difference (moderately explicit stereotype threat; Nguyen & Ryan, 2008). In the control condition (no stereotype activation), the teacher said the learning gains of students who also speak Turkish at home did not differ from that of students who only speak German at home (explicit threat removal strategy; Nguyen & Ryan, 2008). Following the video, participants responded to the psychological scales and finally indicated their sociodemographic data.

The software Unipark (Version EFS_21.2_0164; Tivian XI GmbH) was used for programming the video tutorial and for randomized assignment of participants to the four experimental conditions resulting from the combination of the two independent variables, teacher origin (German vs. Turkish) and stereotype activation (activation vs. no activation).

#### Measures

##### Teacher bias

We used four items to capture the extent to which students thought of the teacher to be unbiased (e.g., “The teacher is unbiased.”; “All students feel encouraged by the teacher, whether they have an immigrant background or not.”). The scale was presented with the following instruction: „Now it’s about the teacher you saw in the video. Imagine that this teacher teaches a class at a secondary school in Berlin. Please give your opinion on the impact of this teacher on her students.“. Participants indicated the extent of their agreement on a five-point Likert-scale (1 = strongly disagree to 5 = strongly agree). The reliability was satisfactory (α = .78).

##### Teacher motivational support

We used four items to assess participants’ perceptions on how motivating the teacher would be for students in general (e.g., “The students feel supported by the teacher.”; “The teacher has a lot of confidence in her students.”). Again, responses were given on a five-point Likert-scale (1 = strongly disagree to 5 = strongly agree). The reliability was good (α = .84).

##### Self-reported family language

Family language was operationalized in two steps. First, participants were asked to indicate the languages they had learned as a young child (response options: German, Turkish, Arabic, Polish, Russian, or another language). Participants where then asked which language they usually speak with their relatives. This question was answered separately for “parents”, “siblings”, and “other relatives (e.g., grandparents, aunts, uncles)” with response options being (1) German only, (2) a language other than German only, (3) both German and another language. Participants were categorized as having a German family language if they chose response option (1). If they indicated that they spoke language/s other than German (response options 2 or 3) with these groups they were categorized as having a Turkish family language if they had already learned Turkish as a child (exclusively or in combination with any other language/s) and as having another non-German family language if they had already learned a language other than Turkish or German as a child (exclusively or in combination with any other language/s).

### Results

Table 1 depicts participants' distribution across the experimental conditions as well as means and standard deviations for teacher bias and teacher motivational support.

**Table 1:** Cell distributions across experimental conditions as well as means and standard deviations for the perception of teacher bias and teacher motivational support in Study 1

Stereotype activation	Teacher origin	Preservice students’ family language	*n*	Teacher bias		Teacher motivation support	
				*M*	*SD*	*M*	*SD*
Yes	German	German	72	2.72	0.98	3.17	0.82
		Turkish	11	2.43	0.86	2.89	0.92
		Other	36	2.82	0.96	3.17	0.71
	Turkish	German	79	3.50	0.93	3.65	0.72
		Turkish	14	3.32	1.10	3.34	0.88
		Other	25	3.52	1.03	3.73	0.94
No	German	German	80	3.72	0.78	3.90	0.69
		Turkish	11	3.50	0.73	3.52	0.89
		Other	23	3.69	0.74	3.84	0.60
	Turkish	German	80	3.88	0.63	4.08	0.70
		Turkish	14	3.93	0.68	3.96	0.66
		Other	23	3.97	0.69	3.82	0.94

To examine preservice teachers’ perceptions of the teacher shown in the video, a multivariate analysis of variance (MANOVA) was computed in SPSS (IBM SPSS Statistics version 28.0.0.0), with the experimental manipulations (teacher origin, stereotype activation) and participants’ language groups (German, Turkish, other language) as between-participant factors. The two scales, teacher bias and teacher motivational support, were included simultaneously to control for correlations between dependent variables. To account for multicollinearity, we computed the bivariate correlation between the two dependent variables. While teacher bias and teacher motivational support were strongly correlated at *r* = .71, the coefficient was below the cutoff criterion for multicollinearity (*r* > .85; Schroeder et al., 1990). It seems that, in preservice teachers' perceptions, the extent to which a teacher is unbiased toward immigrant students is highly correlated with the extent to which she is motivationally supportive of students in general. For post-hoc tests, Bonferroni correction was applied. η^2^ (Cohen, 1988) was calculated as an effect size measure.

#### Perceived teacher bias

There was a main effect of teacher origin (Table 2). As predicted by hypothesis *H1a*, participants perceived the German origin teacher as more biased toward minority students (*M* = 3.15, *SE* = .08) than the Turkish origin teacher (*M* = 3.69, *SE* = .07), *F*(1,456) = 27.41, *p* < .001, η^2^ = .06. Further, as expected in hypothesis *H2a*, the two-way interaction between teacher origin and stereotype activation was significant, *F*(1,456) = 5.75, *p* = .017, η^2^ = .01. Bonferroni-corrected post-hoc comparisons revealed a pattern that was largely consistent with hypothesis *H2a*: Both the German origin teacher, *p* < .001, *M*_Diff_ = .98, 95% CI [.68, 1.27], and the Turkish origin teacher, *p* < .001, *M*_Diff_ = .48, 95% CI [.20, .76], were perceived as more biased when they referred to differences between students speaking only German or also Turkish at home (German teacher: *M* = 2.66, *SE* = .10; Turkish teacher: *M* = 3.45, *SE* = .10), compared to when they did not (German teacher: *M* = 3.63, *SE* = .11, Turkish teacher: *M* = 3.93, *SE* = .10). However, while the Turkish origin teacher (*M* = 3.63, *SE* = .11) and the German origin teacher (*M* = 3.93, *SE* = .10) were both perceived as relatively unbiased when they said that students who spoke German versus also Turkish at home did not differ on the test, *p* = .049, *M*_Diff_ = .29, 95% CI [.00, .59], the German origin teacher was more strongly devalued for referring to a difference between the language groups (*M* = 2.66, *SE* = .10) than the Turkish origin teacher (*M* = 3.45, *SE* = .10), *p* < .001, *M*_Diff_ = .79, 95% CI [.51, 1.07].

**Table 2:** MANOVA on the perception of teacher bias and teacher motivational support, according to experimental conditions (teacher origin, stereotype activation) and preservice teachers’ family language in Study 1

	*F*	*p*	η^2^
Intercept			
Bias	4364.01	< .001***	.91
Motivational support	6081.22	< .001***	.93
Participants’ family language^a^			
Bias	.98	.378	.00
Motivational support	2.78	.063	.01
Teacher origin^b^			
Bias	27.41	< .001***	.06
Motivational support	14.41	< .001***	.03
Stereotype activation^c^			
Bias	49.67	< .001***	.10
Motivational support	33.28	< .001***	.07
Participants’ family language × Teacher origin			
Bias	.25	.776	.00
Motivational support	.23	.798	.00
Participants’ family language × Stereotype activation			
Bias	.20	.820	.00
Motivational support	.81	.444	.00
Teacher origin × Stereotype activation			
Bias	5.75	.017*	.01
Motivational support	2.58	.109	.01
Participants’ family language × Teacher origin × Stereotype activation			
Bias	.14	.868	.00
Motivational support	.66	.515	.00

*N* = 468. *Bias: R*^2^ = 0.225 (adjusted *R*^2^ = 0.207). Motivational support: *R*^2^ = 0.176 (adjusted *R*^2^ = 0.156)

^a^1 = German family language, 2 = Turkish family language, 3 = other family language than German or Turkish

^b^0 = German origin teacher, 1 = Turkish origin teacher

^c ^0 = No-Stereotype threat, 1 = Stereotype threat

*p ≤ .05, **p ≤ .01, ***p ≤ .001

We additionally obtained a main effect of stereotype activation. The teacher was judged as more biased if she referred to differences between students speaking German or also Turkish at home (stereotype activation; *M* = 3.05, *SE* = .07) than if she did not (no stereotype activation; *M* = 3.78, *SE* = .04), *F*(1,456) = 49.67, *p* < .001, η^2^ = .10. No other significant effects were observed.

#### Perceived teacher motivational support

Depicted in Table 2, as predicted by our hypothesis *H1b*, participants perceived the Turkish origin teacher as more motivationally supportive (*M* = 3.76, *SE* = .06) than the German origin teacher (*M* = 3.41, *SE* = .07), *F*(1,456) = 14.41, *p* < .001, η^2^ = .03. Hypothesis *H2b* could not be confirmed: While the pattern of means was consistent with our expectation (stereotype activation: German teacher *M* = 3.07, *SE* = .09; Turkish teacher *M* = 3.57, *SE* = .09; no stereotype activation: German teacher *M* = 3.75, *SE* = 1.00, Turkish teacher *M* = 3.96, *SE* = .09) the interaction effect of teacher origin and stereotype activation did not reach statistical significance (*p* = .109). Rather, we observed a main effect for stereotype activation: The teacher was perceived as less motivationally supportive if she said that Turkish and German speaking students differed on the test (*M* = 3.32, *SE* = .06) than if she said that they did not differ (*M* = 3.85, *SE* = .07), *F*(1,456) = 33.28, *p* < .001, η^2^ = .07. No other effects were statistically significant.

### Discussion

As predicted, irrespective of their own ethnic or cultural background, preservice teachers perceived the Turkish origin teacher to be less biased toward immigrant students and more motivationally supportive of students in general than the German origin teacher. Our expectation that preservice teachers' perceptions of the minority teacher would not be negatively affected when she referred to differences between students of different language groups was met only in terms of teacher bias, but not in terms of teacher motivational support: while the immigrant teacher was not implied to be biased when addressing difficulties of her group, even in the case of the minority teacher were our participants concerned that pointing out group differences in learning gains would have a demotivating effect on school students.

It will be interesting to see in our next study whether school students who may be negatively affected by stereotypes expressed by a teacher experience identity-safety to a different extent, depending on the teacher's background and depending on whether the teacher voicing a stereotype about their language group.

## Study 2

In Study 2, we examined (1) how school students perceived the teacher in the four experimental conditions and (2) how the experimental manipulations affect ethnic minority students' learning.

## Research hypotheses

The first two research hypotheses are completely analogous to the two hypotheses we have specified for preservice teachers in Study 1.

### Hypothesis 1

School students perceive the Turkish origin teacher to be *(H1a)* less biased toward immigrant students' needs, and *(H1b)* more motivating for all students than the majority German origin teacher.

### Hypothesis 2

School students perceive the German origin teacher as more biased *(H2a)* and less motivating *(H2b)* if she addresses the stereotype (compared to when she does not activate the stereotype), while the perception of the Turkish origin teacher is unaffected by her addressing or not addressing the stereotype.

We further explored, with no directional hypotheses, whether school students with German, Turkish, or other non-German family language would differ in their perception of the teacher shown in the videos.

### Hypothesis 3

Students with Turkish family language and ethnic minority students of other non-German family languages suffer in their learning gains in the vocabulary test when the German origin teacher addresses the stereotype (compared to when she does not activate the stereotype), while their learning gains are unaffected by the Turkish origin teacher addressing or not addressing the stereotype *(H3)*.

Without directional hypothesis, we further investigated whether teacher perception (bias and motivational support) had an effect on immigrant students' learning gains.

Regarding students' learning gains in the vocabulary test, we did not expect our experimental treatments to have an effect on students with German family language. Thus, we only explored whether minority students profit from minority teachers in their learning.

### Method

#### Research participants

Our sample comprised 618 students nested in 32 ninth or tenth grade classes from five high schools of upper (“Gymnasium”, from which students graduate with eligibility for university studies) or lower (“Integrierte Sekundarschule”, from which students leave with the eligibility for vocational education and training) academic track in a large city in Germany. Five hundred and ninety-one students gave consent to their use of data. We excluded students who completed the survey in an unrealistic time (-2 *SD*), students with outlying learning gains (±2 *SD*, *n* = 23), students who entered a wrong four-digit code for treatment-assignment (see experimental design and procedure; *n* = 22), and—as we wanted to include gender as a control—students self-identifying as diverse (*n* = 19).

Our final dataset consisted of 527 students. The mean age was 14.88 years (*SD* = .85). Gender was distributed nearly equally, with 242 students identifying as female (45.9%) and 261 as male (49.5%; 24 missing values). One hundred ninety-seven students spoke exclusively German at home (37.4%), 127 also spoke Turkish (24.1%) and 203 (38.5%) also a language other than German or Turkish at home. Among the students with a family language other than German or Turkish, 53 indicated they spoke Arabic, 20 Polish, 30 Russian, and 100 other languages.^4^ Three hundred and twenty-four students attended lower track schools (61.5%; language groups: 25.9% German, 33.0% Turkish, 41.0% other languages) and 203 upper track schools (38.5%; language groups: 55.7% German, 9.9% Turkish, 34.5% other languages).

#### Experimental design and procedure

Data was collected as part of a research project funded by the German science foundation, approved by the School Senator of Berlin and the Ethics Committee of Freie Universität Berlin. School principals were informed about the research project by letter and telephone contact and asked to participate with their classes. As an incentive for participation, each school class received 50 Euros. We chose a pretest–posttest experimental control group design to test our hypotheses. The study was conducted in the classroom. After a brief standardized instruction by trained administrators, students were each given a tablet and completed a tutorial individually, consisting of a learning task and a questionnaire.

The software LimeSurvey (Version 3.28.0; LimeSurvey GmbH, 2015) was used for programming the video tutorial and for randomized assignment to the four experimental conditions resulting from the combination of the two independent variables, teacher origin (German vs. Turkish) and stereotype activation (activation vs. no activation). To ensure that immigrant students related the stereotype to their own language group, we varied the teacher's statement in the experimental conditions so that the stereotype referred either to Turkish-speaking students or to students speaking a language other than Turkish or German (for details see manipulation of stereotype activation below). It was therefore necessary to consider participants’ family language when assigning them to the experimental conditions. We asked teachers in the run-up to the study to allocate a code to each student. Teachers received a list of codes, with each code consisting of four digits: one digit for the family language of the respective student (German; Turkish; other than German or Turkish) and three random digits intended to mask the differentiation by language for the students. Immediately prior to the start of the study, the teacher handed each student his or her code. By entering the code into the tablet, LimeSurvey randomly started one of the four experimental conditions adapted to the student’s family language group.^5^

**Fig. 1:**
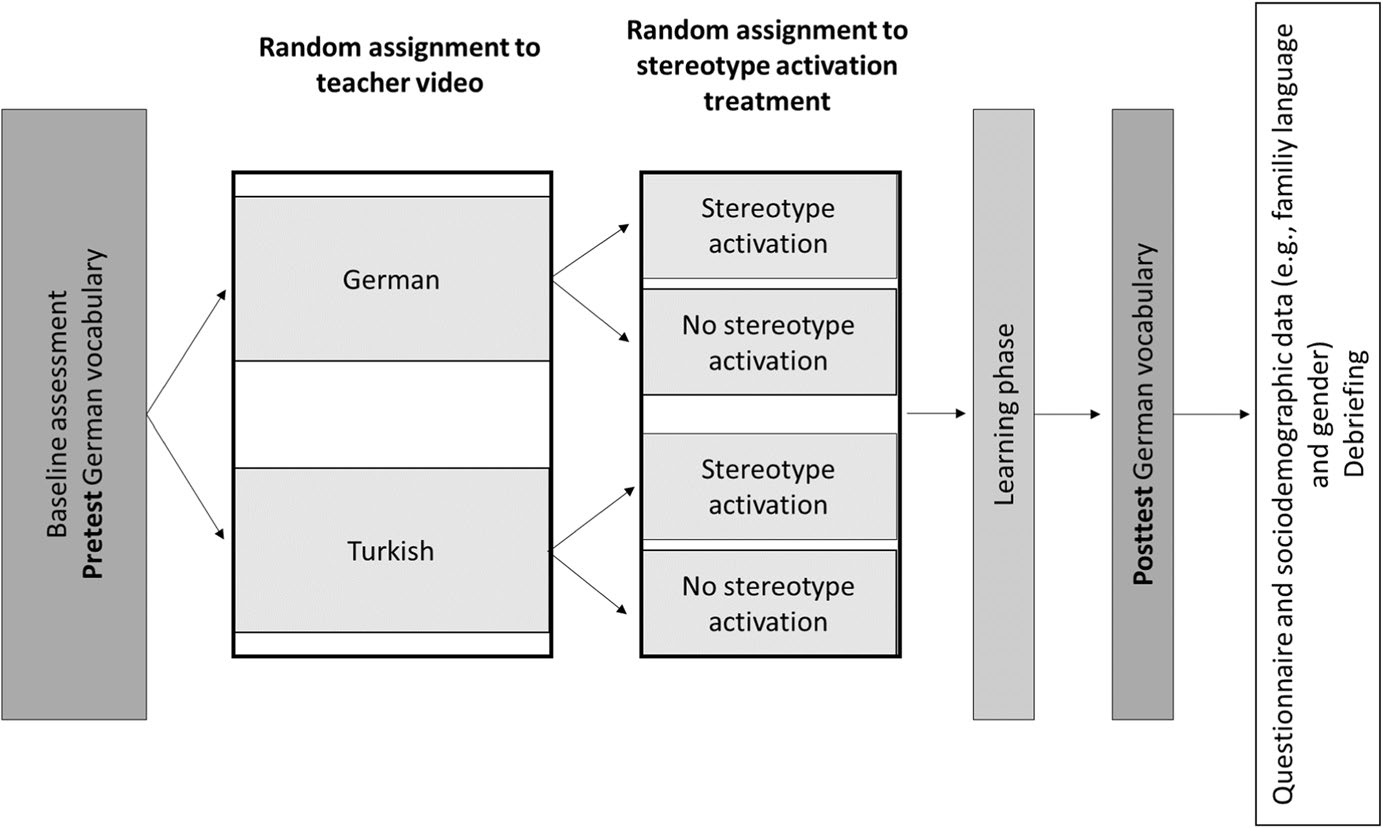
Design and procedure of Study 2

Figure 1 shows the design and procedure which was implemented on the tablets. After students gave consent to the use of their data, they were guided through a tutorial on their tablet by a female teacher. The teacher provided a detailed explanation of the pretest, a vocabulary test in which for a total of 15 difficult target words as well as two icebreaker words (15 + 2) the student had to each time select a synonym word from a list of five options. The pretest was followed by the experimental manipulations (see below for details). In the subsequent learning phase, students were supposed to study the 15 words from the pretest. For each word, they saw a fictitious dictionary entry explaining it, together with a sentence containing the word and illustrating its meaning. The student then had to complete 15 + 2 cloze sentences by selecting one of the previously learned 15 + 2 words (each time from a list of eight). Afterwards, the student was given feedback on the correct answer. The learning phase was followed by the posttest where—like in the pretest—the 15 + 2 words were presented and the student had to select the appropriate synonym from a list of five words. The tutorial concluded with a questionnaire containing our dependent variables and a debriefing of the purpose of the study.

##### Manipulation of teacher origin

Teacher origin was manipulated in the same way as in Study 1.

##### Manipulation of stereotype activation

Stereotype activation was manipulated in the same way as in Study 1 for students with German and Turkish family language. For students with other non-German family language, however, the treatment was adapted. Here, the teacher said that on the vocabulary test learning success of adolescents who speak only German at home differed (stereotype activation)/did not differ (no stereotype activation) from the learning success of adolescents who also speak “a language other than German at home”.

#### Measures

##### Vocabulary pretest and posttest

Students’ responses in the pretest and posttest were coded dichotomously (0 = not correct, 1 = correct), resulting in a maximum of 15 points for the respective test. Reliabilities were satisfactory for the pretest (α = .67) and posttest (α = .85).

##### Teacher bias

Students’ perceptions of teacher bias was measured using the same four items as in Study 1. The reliability was acceptable ($\upomega$ = .69).^6^

##### Teacher motivational support for all students

Students’ perceptions of motivational support was measured using the same four items as in Study 1, however, the wording was adapted to students’ perspective (e.g., “I feel supported by the teacher”). The reliability was good ($\upomega$ = .83).

##### Self-reported family language

Family language was measured in the same way as in Study 1.

##### Gender

Since boys may be additionally affected by negative stereotypes about their gender group in language domains (Li & McLellan, 2021), we controlled for students’ gender in our analyses. Since students who self-identified as diverse were excluded, we considered binary gender in our analyses. Gender was recorded as a dummy variable (0 = girls, 1 = boys).

##### Socioeconomic status

Students’ socioeconomic status was determined by the number of books in the household, adopted from Mang et al. (2018): “If you think about it: How many books do you have in your home? (Note: You can fit about 40 books on one meter of shelf space)”. Responses were given on a seven-point scale (response options:1 = none, 7 = more than 500).

##### School type

To account for differences in learning gains due to school track, we included a dummy variable for type of school (0 = lower academic track, 1 = upper academic track).

##### Self-reported grades in German

Self-reported grades in the school subject German were included to account for students’ verbal competences. The students reported their last grade on their school report on a scale from 1 to 6 which was inverted (6 = “excellent” to 1 = “insufficient”).

##### Academic self-concept in the school subject German

We additionally considered motivational characteristics as source of success in the word learning task. Hence, we also assessed the academic self-concept, which has been associated with adaptive learning behaviors (Marsh & O’Mara, 2008; Trautwein & Möller, 2016) and positive learning and achievement outcomes (Marsh & Craven, 2006). We used a German short version by Kunter et al. (2002) of the Self-Description Questionnaire by Marsh (1990) consisting of three items on coping with academic demands in the school subject German (e.g., “In the subject German I learn quickly.”). Answers were given on a five-point Likert-scale (1 = strongly disagree to 5 = strongly agree). The reliability was good ($\upomega$ = .83).

### Results

Table 3 shows the cell distributions of participants across the experimental conditions, means and standard deviations for teacher bias as well as teacher motivational support.

**Table 3:** Cell distributions across experimental conditions as well as means and standard deviation for the perception of teacher bias and teacher motivational support in Study 2

Stereotype activation	Teacher origin	School students’ family language	*n*	Teacher bias		Teacher motivation support	
				*M*	*SD*	*M*	*SD*
Yes	German	German	46	3.67	0.85	3.39	0.96
		Turkish	25	3.50	0.83	3.09	0.99
		Other	35	3.20	0.89	3.11	0.95
	Turkish	German	60	3.63	0.80	3.55	0.84
		Turkish	34	3.54	0.95	3.50	1.10
		Other	71	3.55	0.78	3.36	0.90
No	German	German	55	3.70	0.85	3.32	0.93
		Turkish	28	3.01	1.13	3.21	1.17
		Other	45	3.72	0.75	3.46	1.03
	Turkish	German	36	3.74	1.01	3.29	0.92
		Turkish	40	3.35	0.96	3.31	1.02
		Other	52	3.51	0.83	3.19	0.95

In analogy to our approach in Study 1, we examined school students’ perceptions of the teacher (bias and motivational support) via a multivariate analysis of variance (MANOVA) with the experimental manipulations (teacher origin, stereotype activation) and participant’s language groups (German, Turkish, other language) as between-participant factors. The analysis was computed using SPSS (IBM SPSS Statistics version 28.0.0.0). With *r* = .66, the bivariate correlation between the two dependent variables was below the cutoff criterion for multicollinearity (Schroeder et al., 1990). While the correlation was weaker than for preservice teachers in Study 1, school students also thought of the teacher as the more motivationally supportive of all students the more they considered her as unbiased toward immigrant students. As students were nested within 32 school classes, we explored whether we needed to control for the nested data structure. The intraclass correlation coefficient (ICC) indicated that 3% and 0.3% of the variance in school students’ perceptions of teacher bias and teacher motivational support respectively were explained by the classroom level. We therefore did not have to consider the nested data structure separately in our analyses as controlling for data clusters is recommended for ICC > .05 (Julian, 2001).

#### Perceived teacher bias

Table 4 shows the results of the replicated MANOVA of Study 1 for our school student sample. Other than predicted by our hypotheses, there was no main effect of teacher origin *(H1a)* and no interaction effect of teacher origin and stereotype activation *(H2a)*.

**Table 4:** MANOVA on the perception of teacher bias and teacher motivational support, according to experimental conditions (teacher origin, stereotype activation) and school students’ family language in Study 2

	*F*	*p*	η^2^
Intercept			
Bias	7814.30	< .001***	.94
Motivational support	5701.04	< .001***	.92
Participants’ family language^a^			
Bias	5.83	.003**	.02
Motivational support	.73	.482	.00
Teacher origin^b^			
Bias	1.20	.275	.00
Motivational support	1.41	.236	.00
Stereotype activation^c^			
Bias	.02	.878	.00
Motivational support	.16	.687	.00
Participants’ family language × Teacher origin			
Bias	.46	.632	.00
Motivational support	.71	.492	.00
Participants’ family language × Stereotype activation			
Bias	4.18	.016*	.02
Motivational support	.80	.448	.00
Teacher origin × Stereotype activation			
Bias	.14	.711	.00
Motivational support	3.81	.052	.01
Participants’ family language × Teacher origin × Stereotype activation			
Bias	2.73	.066	.01
Motivational support	.33	.720	.00

*N* = 527. *Bias: R*^2^ = 0.046 (adjusted *R*^2^ = 0.026). Motivational support: *R*^2^ = 0.019 (adjusted *R*^2^ = -0.002)

^a ^1 = German family language, 2 = Turkish family language, 3 = other family language than German or Turkish

^b ^0 = German origin teacher, 1 = Turkish origin teacher

^c ^0 = No-Stereotype threat, 1 = Stereotype threat

*p ≤ .05, **p ≤ .01, ***p ≤ .001

A main effect of students' family language appeared, *F*(1,527) = 5.83, *p* = .003, η^2^ = .02. Bonferroni-corrected post-hoc analysis indicated that students with German family language were less concerned that the teacher was biased toward minority students (*M* = 3.69, *SE* = .06) than students with Turkish family language (*M* = 3.35, *SE* = .08), *p* = .003, *M*_Diff_ = .34, 95% CI [.09, .58], and then—while only marginally significantly so—students with other non-German family language (*M* = 3.50, *SE* = .06), *p* = .092, *M*_Diff_ = .19, 95% CI [− .02, .41]. Further, we observed a significant two-way interaction between students' family language and stereotype activation, *F*(2,527) = 4.18, *p* = .016, η^2^ = .02. Bonferroni-corrected post-hoc comparisons revealed the following pattern: When the teacher stated there was no difference in learning gains (no stereotype activation), students with Turkish family language were more concerned that the teacher was biased toward minority students (*M* = 3.18, *SE* = .11) than the students with German (*M* = 3.72, *SE* = .09), *p* < .001, *M*_Diff_ = .54, 95% CI [.20, .88], and other non-German family language (*M* = 3.61, *SE* = .09), *p* = .006, *M*_Diff_ = .43, 95% CI [.09, .77]. In contrast, when the teacher referred to differences in learning gains (stereotype activation) students' perceptions of the teacher did not differ between language groups. When comparing the two stereotype activation conditions within each of the three language groups, this pattern of means resulted in a significant difference only for the Turkish group: Students with Turkish family language perceived the teacher as less biased when she referred to differences between language groups (stereotype activation: *M* = 3.52, *SE* = .12) compared to when she said there were no differences between language groups (no stereotype activation: *M* = 3.18, *SE* = .11), *p* = .030, *M*_Diff_ = .34, 95% CI [.03, .65]. No other significant effects were observed.

#### Perceived teacher motivational support

As depicted in Table 4, we found the expected interaction effect between teacher origin and stereotype activation *(H2b)*, it was, however, only marginally significant, *F*(1,527) = 3.81, *p* < .052, η^2^ = .01. As predicted, under stereotype activation the German origin teacher was perceived as less motivationally supportive (*M* = 3.20, *SE* = .10) than the Turkish origin teacher (*M* = 3.47, *SE* = .08), *p* = .028, *M*_Diff_ = .28, 95% CI [.03, .52] while there was no difference in perception of motivational support between the teachers when no stereotype was activated (German origin teacher: *M* = 3.33, *SE* = .09; Turkish origin teacher: *M* = 3.27, *SE* = .09), *p* = .587, *M*_Diff_ = .07, 95% CI [− .18, .31]. No other significant effects were observed.

#### Immigrant students' learning gains

Next, we examined the effects of our experimental manipulations on students' posttest scores in the vocabulary learning task. As already mentioned, to keep our models as parsimonious as possible we excluded German origin students from our multiple-group regression analysis.^7^ As only immigrant students were expected to potentially feel threatened by the teacher referring to differences between language groups in their learning success, we considered the subsamples of 330 students with Turkish family language (*n* = 127) or other non-German family language (*n* = 203) only. In total, 22% percent of students' pretest scores and 33% of their posttest scores were explained via classroom level, thus we had to consider the classroom level in all further analyses. To account for this nested data structure and to investigate students' posttest score via multiple group analysis, we used Mplus version 8.7 (Muthén & Muthén, 2017) with the TYPE = COMPLEX command correcting for underestimated standard errors of our model parameters. Coefficients were estimated using Robust Maximum Likelihood (MLR). Missing values for single items were accounted for by the Full Information Maximum Likelihood (FIML). We included all our multi-item measures (academic self-concept in the school subject German, teacher bias and teacher motivational support) as latent constructs into our regression models.

##### Measurement invariance

A prerequisite condition for group mean comparisons between Turkish and other non-German language students' learning gains are invariant factor patterns, invariant factor loadings, as well as item intercepts are crucial for group mean comparison (Brown, 2006; Vandenberg & Lance, 2000). We therefore first tested the measurement invariance of our latent measurement models (academic self-concept, teacher bias, motivational teacher support). Following van de Schoot et al. (2012), we conducted multiple group analyses by estimating model parameters simultaneously for the two language groups using the GROUPING command in Mplus in various invariance models (see Table 5). In our first model, we assumed an invariant factor pattern across groups. This model was expanded stepwise, by additionally constraining invariant factor loadings (M2), invariant factor loadings, and invariant item intercepts (M3), and finally invariant factor loadings, item intercepts, and item uniqueness (M4). According to Cheung and Rensvold (2002) as well as Chen (2007), measurement invariance can be assumed when changes in the CFI and TLI do not drop more than .01 and the RMSEA does not change more than .015.

**Table 5:** Goodness-of-fit indices for the measurement model for students with Turkish and other non-German family language in Study 2

Model	Invariance	χ^2^	*df*	RMSEA (90% CI)	SRMR	TLI	CFI
M1	Configural	115.919*	76	.056 (.034 – .076)	.045	.945	.962
M2	Metric	127.506*	87	.053 (.032 – .072)	.083	.951	.961
M3	Scalar	143.475*	98	.053 (.033 – .071)	.089	.951	.957
M4	Strict	144.258	109	.044 (.021 – .063)	.091	.966	.966

*χ^2^ values were significant (*p* ≤ .05)

First, we specified a confirmatory factor analysis that separately assessed the theoretically operationalized constructs for students with Turkish family language (χ^2^ [df = 38, *N* = 127] = 63.611; p = .006; CFI = .950; TLI = .927; RMSEA = .073; SRMR = .049) and students with other non-German family language (χ^2^ [df = 38, *N* = 203] = 52.657; p = .057; CFI = .973; TLI = .961; RMSEA = .044; SRMR = .042), and a multiple group analysis of configural invariance assuming the same factor structure for both groups (Table 5, Model 1).

Considering the range of information, including factor loadings, descriptive goodness-of-fit indices, and changes between models, we achieved strict invariance (M4) with a ∆RMSEA < .015 and ∆CFI < − .01, although the better fit for the SRMR is found in the scalar model (M3). In any case, we can draw valid conclusions about factor means in our analyses.

##### Descriptive statistics and intercorrelations

Next, we calculated descriptive statistics for students with Turkish family language and students with other non-German family language separately. The means and standard deviations as well as intercorrelations for students with Turkish and other non-German family language are documented in Table 6. Descriptively, students with Turkish family language were found to have, on average, both lower pretest scores (*M* = 2.82, *SD* = 1.79) and lower posttest scores (*M* = 6.35, *SD* = 3.64) than students of other non-German family language (Pretest: *M* = 3.91, *SD* = 2.60; Posttest *M* = 7.78, *SD* = 4.18). Background characteristics and academic self-concept were similar on average in both groups. As already evident in the MANOVA (see Table 4), students with Turkish family language perceived the teacher as more biased toward minority students' needs compared to students with other family language, while students of both language groups did not differ in their perception of motivational support from the teacher.

**Table 6:** Means, standard deviations, and correlations for students with Turkish family language (below) and students with other non-German family language (above) in Study 2

Variable	*M*_*Turkish*_ (*SD*)	*M*_*Other*_ (*SD*)	1	2	3	4	5	6	7	8	9	10	11
1. Posttest	6.35 (3.64)	7.78 (4.18)	–	.72***	-.13	.41**	.53***	.21**	.07	-.12*	-.19*	.17*	-.06
2. Pretest	2.82 (1.79)	3.91 (2.60)	.52***	–	-.07	.41***	.48***	.22**	.13	-.08	-.22**	.06	-.13
3. Gender^a^	.46 (.50)	.48 (.50)	-.07	.00	–	.04	-.05	− .14*	-.05	.03	.02	-.14	-.09
4. SES	3.28 (1.50)	3.25 (1.48)	.21	.13	.155*	–	.47***	.13	.14**	-.05	-.17*	-.02	-.11
5. Type of school^c^	.16 (.36)	.35 (.48)	.47***	.26**	-.14	.30*	–	.00	.15	.03	-.33**	.08	-.10
6. German grade^b^	3.91 (.96)	3.91 (.94)	.13	.01	-.08	.12	-.09	–	.64***	-.05	.10	.19*	.12
7. Academic self-concept^f^	3.28 (.84)	3.31 (.68)	.16	-.16	.02	.03	-.09	.37**	–	-.06	.17*	.28*	.33**
8. Stereotype activation^d^	− .04 (.50)	.02 (.50)	.05	.12	-.12	-.12	.07	-.05	.08	–	.14*	-.14	-.02
9. Origin of the teacher^e^	.58 (.49)	.61 (.49)	-.11	-.12	-.06	-.06	-.16	.03	-.03	-.01	–	.02	.01
10. Teacher bias^f^	3.28 (.97)	3.54 (.81)	.34***	.13	− .11	.12	.12	.12	.50***	.18	.09	–	.86***
11. Teacher motivational support^f^	3.43 (1.08)	3.38 (1.00)	.09	-.07	-.11	.05	-.07	.06	.45***	.06	.11	.94***	–

*N*_Turkish_ = 127, *N*_*other*_ = 203. All values were estimated using Mplus and missing values were accounted for using FIML

^a^0 = girls, 1 = boys

^b^Grades are inverted from 6 = “excellent” to 1 = “insufficient”

^c^ 0 = lower academic track, 1 = upper academic track

^d^-0.5 = no stereotype activation, 0.5 = stereotype activation

^e ^0 = German origin teacher, 1 = Turkish origin teacher

^f^ Results based on latent scale scores

*p ≤ .05, **p ≤ .01, ***p ≤ .001

For students with a different non-German family language, the intercorrelations further showed that the experimental treatments, stereotype activation and teacher origin, were each negatively correlated with their posttest scores: These students learned fewer words when the teacher said that their ingroup differed in their learning gains from students with German family language than when the teacher said there was no such difference, and they learned fewer words when they were guided through the learning task by the Turkish origin teacher rather than the German origin teacher. In contrast, the corresponding correlations for Turkish students were not statistically significant. For both groups, the perception of teacher bias was positively related to students' posttest scores, meaning that students achieved a higher posttest score the more they perceived the teacher to be unbiased. Perceptions of how motivationally supportive the teacher is did not correlate with posttest scores in either group.

##### Results from stepwise regression models

We computed stepwise multiple-group regression models predicting German vocabulary acquisition for students with Turkish family language and for students of other non-German language. To reduce the complexity of the models due to the experimental design, we ran our models separately for the German and Turkish origin teacher. In Model 1, we regressed students' posttest scores on stereotype activation (no activation = 0.5, activation = 0.5), controlling for pretest scores as well as our control variables of socioeconomic status, gender, German grade, type of school, and academic self-concept. In Model 2, we added teacher bias and in Model 3 teacher motivational support.

Tables 7 and 8 show the results of the stepwise multiple-group regression analyses for immigrant students instructed by a German and Turkish origin teacher, respectively. Our regression models indicated sufficient model fit. The results for our controls were as follows: For students instructed by the German origin teacher, only pretest score and school type were associated with posttest score, indicating that students attending schools of higher academic track learned significantly more vocabulary than students attending school of lower academic track. In addition, when instructed by a German origin teacher, the academic self-concept was predictive for the posttest score of students with Turkish family language (β = .30, *p* = .008), meaning the higher the academic self-concept, the better these students performed on the posttest.

**Table 7:** Results from stepwise multiple-group regression analysis predicting posttest scores for immigrant students instructed by the German origin teacher in Study 2

Student’s family language	M1						M2						M3					
	Turkish			Other Non-German			Turkish			Other Non-German			Turkish			Other Non-German		
	*B* *(SE)*	β	*p*	*B* *(SE)*	β	*p*	*B* *(SE)*	β	*p*	*B* *(SE)*	β	*p*	*B* *(SE)*	β	*p*	*B* *(SE)*	β	*p*
Pretest	1.12 (0.17)	.55	< .001***	1.00 (0.11)	.62	< .001***	1.09 (0.16)	.53	< .001***	1.00 (0.11)	.62	< .001***	1.12 (0.17)	.55	< .001***	1.00 (0.11)	.62	< .001***
Gender^a^	0.05 (0.59)	.01	.936	0.01 (0.62)	.00	.994	0.22 (0.65)	.03	.728	-0.01 (0.60)	.00	.987	0.12 (0.64)	.02	.852	0.08 (0.60)	.01	.893
SES	.09 (0.28)	.03	.738	0.33 (0.20)	.12	.088	0.05 (0.31)	.02	.881	0.35 (0.19)	.13	.056	0.09 (0.28)	.03	.735	0.35 (0.19)	.13	.057
School type^b^	4.71 (0.76)	.51	< .001***	1.84 (0.85)	.22	.025*	4.43 (0.80)	.48	< .001***	1.80 (0.79)	.21	.019*	4.73 (0.74)	.51	< .001***	1.85 (0.74)	.22	.010*
German grade^c^	0.19 (0.30)	.05	.532	0.23 (0.52)	.05	.663	0.21 (0.25)	.06	.405	0.18 (0.41)	.04	.670	0.22 (0.28)	.06	.416	0.31 (0.37)	.07	.414
Academic self-concept	1.72 (0.17)	.30	.008**	0.20 (1.16)	.02	.865	1.29 (0.59)	.26	.025*	0.16 (0.72)	.02	.818	1.54 (0.55)	.30	.006**	-0.02 (0.61)	.00	.969
Stereotype activation^d^	0.03 (0.87)	.00	.973	0.27 (0.54)	.03	.615	-0.11 (0.93)	− .01	.903	0.33 (0.60)	.04	.588	0.03 (0.86)	.00	.970	0.33 (0.56)	.04	.564
Teacher bias							0.43 (0.50)	.11	.405	0.16 (0.39)	.03	.683						
Teacher motivational support													-0.06 (0.36)	-.02	.878	0.23 (0.27)	.06	.420
${R}^{2}$	.65		< .001	.65		< .001	.70		< .001	.65		< .001	.65		< .001	.65		< .001

*N* = *133*

^a ^0 = girls, 1 = boys

^b^ 0 = lower academic track, 1 = upper academic track

^c^ Grades are reversed from 6 = “excellent” to 1 = “insufficient”

^d ^− 0.5 = no stereotype activation, 0.5 = stereotype activation

M1 (χ^2^ [df = 42, N = 133] 31.781; *p* = .874; CFI = 1.000; TLI = 1.000; RMSEA = 0.000; SRMR = .058), M2 (χ^2^ [df = 120, N = 133] = 175.340; *p* = .001; CFI = .883; TLI = .852; RMSEA = .083; SRMR = .094), M3 (χ^2^ [df = 119, N = 133] = 129.228; *p* = .246; CFI = .982; TLI = .977; RMSEA = .036; SRMR = .071. *p ≤ .05, **p ≤ .01, ***p ≤ .001

**Table 8:** Results from stepwise multiple-group regression analysis predicting posttest scores for students instructed by the Turkish origin teacher in Study 2

Student’s family language	M1						M2						M3					
	Turkish			Other Non-German			Turkish			Other Non-German			Turkish			Other Non-German		
	*B* *(SE)*	β	*p*	*B* *(SE)*	β	*p*	*B* *(SE)*	β	*p*	*B* *(SE)*	β	*p*	*B* *(SE)*	β	*p*	*B* *(SE)*	β	*p*
Pretest	0.88 (0.19)	.43	< .001***	0.80 (0.12)	.50	< .001***	0.81 (0.19)	.40	< .001***	0.79 (0.12)	.50	< .001***	0.88 (0.19)	.43	< .001***	0.82 (0.13)	.52	< .001***
Gender^a^	-0.51 (0.75)	-.07	.492	-1.05 (0.57)	-.13	.064	-0.34 (0.76)	-.05	.656	-0.67 (0.42)	-.09	.115	-0.50 (0.72)	-.07	.481	-1.04 (0.56)	-.13	.060
SES	.04 (0.26)	.02	.863	− 0.01 (0.25)	.00	.998	-0.03 (0.26)	-.02	.897	0.16 (0.27)	.06	.551	0.01 (0.23)	.01	.955	0.08 (0.25)	.03	.749
School type^b^	1.94 (1.45)	.17	.219	2.27 (0.85)	.24	.011*	2.34 (1.49)	.21	.159	1.54 (0.81)	.16	.077	2.12 (1.44)	.19	.184	2.04 (0.88)	.22	.026*
German grade^c^	0.55 (0.38)	.13	.151	0.49 (0.43)	.12	.261	0.70 (0.38)	.17	.069	0.69 (0.32)	.17	.037*	0.65 (0.42)	.15	.122	0.65 (0.32)	.15	.053
Academic self-concept	0.87 (0.62)	.19	.182	-0.28 (0.85)	-.05	.742	0.23 (0.63)	.06	.720	-1.03 (0.77)	-.19	.194	0.67 (0.61)	.16	.285	-0.76 (0.66)	-.14	.258
Stereotype activation^d^	-0.75 (0.65)	-.11	.254	-1.24 (0.52)	-.16	.019*	-0.77 (0.66)	-.11	.247	-1.18 (0.50)	-.15	.020*	-0.81 (0.64)	-.12	.210	-1.25 (0.51)	.16	.018*
Teacher bias							0.68 (0.43)	.21	.110	1.22 (0.57)	.25	.014*						
Teacher motivational support													0.22 (0.39)	.07	.569	0.35 (0.35)	.08	.323
${R}^{2}$	.37		< .001	.52		< .001	.40		< .001	.57		< .001		.38	< .001		.53	< .001

*N* = *197*

^a ^0 = girls, 1 = boys

^b^ 0 = lower academic track, 1 = upper academic track

^c^ Grades are reversed from 6 = “excellent” to 1 = “insufficient”

^d ^-0.5 = no stereotype activation, 0.5 = stereotype activation

M1 (χ^2^ [df = 42, N = 197] = 38.288; *p* = .000; CFI = .1.000; TLI = .1.000; RMSEA = .000; SRMR = .051), M2 (χ^2^ [df = 118, N = 197] = 141.831; *p* = .067; CFI = .955; TLI = .942; RMSEA = .045; SRMR = .061), M3 (χ^2^ [df = 119, N = 197] = 134.783; *p* = .153; CFI = .976; TLI = .969; RMSEA = .037; SRMR = .059). *p ≤ .05, **p ≤ .01, ***p ≤ .001

When instructed by the Turkish origin teacher, for students with Turkish family language only the pretest score was predictive for the posttest score. For students with other non-German family language, pretest score as well as type of school positively predicted posttest score. In neither language group was academic self-concept in German predictive of posttest score.

We then tested our hypothesis *H3* according to which students with Turkish or other non-German family language should be impaired in their vocabulary acquisition when the German majority teacher, but not when the Turkish minority teacher, said that their language ingroups differed in their learning gains from students with German family language. Table 7 (Model 1) shows the results of the stepwise multiple-group regression analysis for the students instructed by the German origin teacher. Neither students with Turkish family language (β = .00, *p* = .973) nor students with other non-German family language (β = .03, *p* = .615) were impaired in their posttest score by the stereotype about their group activated by the German origin teacher. Table 8 (Model 1) shows the results for the students who were instructed by the Turkish origin teacher. Again, students with Turkish family language were found to be unaffected by the activation of the negative stereotype about their group in their posttest score (β = -.11, *p* = .254). In contrast, students with other non-German family language acquired significantly less vocabulary when the teacher said that their ingroup's learning gains differed from those of students with German family language than when the teacher said that there was no such difference (β = -.16, *p* = .019; Table 8). Hence, hypothesis *H3* could not be confirmed.

To test whether teacher bias and teacher motivational support had an impact on students’ learning outcomes when a stereotype about their group was activated, we included these variables stepwise into our multiple-group regression models for students who were instructed by the German origin (Table 7) or the Turkish origin teacher (Table 8). As Model 2 in Table 8 shows, for students with other non-German family language instructed by the Turkish origin teacher, perceptions of teacher bias were positively predictive for students' posttest scores (β = .25, *p* = .014). The more students with other non-German family language perceived the Turkish origin teacher as unbiased, the more successful they were in the learning phase which resulted in higher posttest scores independent of the activation of a stereotype. Students with Turkish family language were not affected in their learning gains by their perceptions of teacher bias. The posttest scores of students who were instructed by the German origin teacher (Table 7, Model2) were not related to their perceptions of teacher bias. Additionally, Model 3 in Table 7 and Model 3 in Table 8 show that teacher motivational support did not affect students' posttest scores in any case. Considering the negative effect of stereotype activation on posttest scores of students with other non-German family language, there was no change in effect size after the introduction of the teacher perception variables.

## General Discussion

It is a widely shared assumption that ethnic minority teachers can be particularly supportive of immigrant students (cf. Georgi et al., 2011; Marx et al., 2012; Morgenroth et al., 2015), especially because they can prevent identity-threats posed by negative stereotypes (Steele, 1997). In our research, we aimed to test the assumption that an immigrant teacher is perceived as providing more identity-safety than an ethnic majority teacher, even when expressing a stereotype about immigrant students as a member of the stigmatized ingroup. Although we expected that both preservice teachers and school students would share this view, our results show a different pattern for the two groups.

### Preservice teachers' perceptions of the teacher

Previous research has shown that teachers with an immigrant background (preservice or trainee) have more positive attitudes and stronger self-efficacy toward teaching ethnically diverse classes (Hachfeld et al., 2012; Syring et al., 2019) and stronger multicultural beliefs (Hachfeld et al., 2012) than German majority teachers (preservice or trainee). To complement these findings, our first study aimed to find out how preservice teachers with and without immigrant background perceive teachers with and without immigrant background in terms of the identity-safety they provide for school students when voicing or not voicing a stereotype about immigrant students. Results showed that preservice teachers' perceptions were not affected by their own cultural or ethnic background: participants with German, Turkish, or other cultural backgrounds perceived the ethnic minority teacher as less biased toward immigrant students and more motivating to students than the ethnic majority teacher. Hence, we found no evidence for group-based striving for positive distinctiveness (Tajfel & Turner, 1979).

As we predicted, the expression of the stereotype led to the teacher being perceived as biased if she herself was an ethnic majority, but not if she was an ethnic minority group member. It seems, that the Turkish origin teacher pointing out difficulties of students of her language ingroup was interpreted by our participants as sensitivity and acknowledgement of immigrant students' particular learning needs, whereas the same behavior of a German majority teacher raised the suspicion that she might be biased toward immigrant students. Other than expected, even the minority teacher, as perceived by our preservice teacher participants, demotivated school students by claiming that there were differences in learning gains between majority and minority language groups. Apparently, our participants were concerned that a teacher’s comment that students from different language backgrounds differed in their learning success could trigger stereotype threat in stigmatized students, whether the teacher was a minority member himself or herself. One possible explanation is that participants believed that inequalities are reproduced by making them an issue. Preservice teachers appear to be very sensitive to the potentially negative effects of stereotypes on student motivation, even when voiced by a minority teacher.

### School students' perceptions of the teacher

In our second study, we wanted to find out whether school students who themselves may be affected by stereotypes expressed by a teacher feel more identity-safe when the teacher has an immigrant background herself, in particular when a negative stereotype about their ingroup is voiced. Deviating from what preservice teachers thought, school students' perceptions of teacher bias was unaffected by the teacher's cultural background and unaffected of whether the teacher said that students of minority language groups did or did not differ in their learning gains from students of the German language majority. Also, in contrast to what we found in our preservice teachers, school students' perceptions of the teacher did depend on their own ethnic background: immigrant students, in particular students with Turkish roots, were more concerned than students of the German majority that the teacher was biased toward immigrant students. This is a disturbing finding, suggesting that immigrant students attending schools in Germany assume teachers agree with the negative stereotype that exists in society about their groups (Asbrock, 2010; Froehlich & Schulte, 2019; Stang et al., 2021), and do so even when the teacher is a member of an ethnic minority.

In addition to what we had predicted in our research hypotheses, we found that students of Turkish descent perceived the teacher—irrespective of whether she had Turkish or German origin—as less biased when she said that previous studies had revealed differences in learning gains between students with Turkish versus German family language than when she said that no such difference had been observed. This finding is consistent with the results of Ollrogge et al. (2022). In their study, Turkish origin students learned most when instructed by an ingroup minority teacher who said that students with Turkish family language often had difficulties learning new German vocabulary (compared to when instructed by an ingroup minority teacher who did not refer to difficulties of the stigmatized ingroup or by a German majority teacher voicing or not voicing challenges of the Turkish group). Ollrogge et al. (2022) come to conclude that immigrant students were most motivated when instructed by an immigrant teacher who spoke openly about challenges that might impede the stigmatized ingroup's academic achievements to help students to overcome them as it is the teacher's responsibility to identify problems that might hamper learning. It seems, students with Turkish family language are aware of their group being academically less successful and they expect a teacher to be sensitive toward their being disadvantaged rather than denying or ignoring the difference in learning prerequisites. In our study, students with Turkish family language not only wished for this openness and sensitivity from the Turkish origin teacher, but also from the German origin teacher. Although we did not predict this result, it is consistent with the findings from research on teacher multicultural beliefs (Hachfeld et al., 2012; Schachner, 2019; Schwarzenthal et al., 2018): Stigmatized students do not want the teacher to pretend that there is no disadvantage of students with an immigrant background, but they want the teacher to be sensitive and open toward cultural diversity and different learning prerequisites, even if the teacher does not belong to their stigmatized ingroup. The demand for teacher's sincerity and needs-oriented promotion might also be supported by the high aspirations of Turkish origin students as we measured by academic self-concept. The positive effect of academic self-concept for students with Turkish family language remained even after controlling for achievement and social background and thus points to a specific resource which is known from other studies as well (Hildebrandt, 2014).

Regarding motivation, our second study revealed, as expected, that students felt equally strongly supported by the German or the Turkish origin teacher as long as she said that minority language students did not differ in their learning gains from students with German family language. However, whereas students felt demotivated when the German majority teacher stated that there were differences between language groups their motivation was not impaired when the Turkish minority teacher said so. While the interaction between teacher origin and stereotype activation narrowly fell short of the statistical significance level, this pattern of findings substantiates our assumption that a minority teacher is perceived to provide identity-safety (Cohn-Vargas & Steele, 2016), so stereotype activation need not interfere with students' motivation. This result is also consistent with previous research showing that ingroup expert role models can prevent stereotype threat as they invalidate the negative stereotype about their group (see meta-analysis by Liu et al., 2021).

### Immigrant students' learning gains

Finally, in our second study, we also investigated whether immigrant students were differentially influenced in their learning gains in the vocabulary task by the experimental variation of teacher origin and stereotype activation. We hypothesized that students with Turkish or other non-German family language would suffer from the teacher saying that their group differed in learning gains from students of the German language majority group, but only when no identity-safety was provided by a minority teacher.

Other than expected, our findings differed for the two immigrant student groups. While students with Turkish roots were unaffected by the stereotype activation manipulation, immigrant students with other non-German family language were impaired in learning new vocabulary when the Turkish origin teacher, but not when the German origin teacher said that their learning gains differed from those of students from the German majority language. That we found different effects for students with Turkish family language than for students with other minority family language contradicts the results by Chaney et al. (2018) who reported that both, a member of the stigmatized ingroup and a member of a similarly stigmatized outgroup, prevented stereotype threat effects in stigmatized test takers. The differential pattern of our findings for the two language groups thus contradicts our assumption that the Turkish origin teacher would provide identity-safety and buffer identity-threats for all students sharing her experience of having a migration history.

In summarizing the effects of our stereotype threat manipulation on students' learning, the only effect we found was that students with a family language other than German or Turkish underperformed when the Turkish origin teacher said that their group's learning gains differed from those of students with German family language. This means that for this group of immigrant students, the Turkish origin teacher did not buffer but boost stereotype threat effects. In the following, we discuss possible explanations for this unexpected pattern of results.

#### Learning gains in Turkish origin students

Why did students with Turkish family language not underperform when the teacher said that students with Turkish family language differed in their learning gains from students with German family language? By the teacher not mentioning the direction of the difference between language groups, we have used a “moderately explicit cue” to induce threat (Nguyen & Ryan, 2008). By the teacher claiming there was no difference between language groups in the control condition, we used an “explicit threat-removal strategy” (Nguyen & Ryan, 2008). Possibly, our two experimental conditions were thus too similar to differentially impact students' learning. So, it is conceivable that in both conditions, the mental accessibility of the stereotype about people of Turkish descent (Asbrock, 2010; Froehlich & Schulte, 2019; Stang et al., 2021) was increased by the very fact that the teacher compared the two language groups. As Turkish origin students can be assumed to be aware that the stereotype about their group is negative, the mere comparison with the ethnic majority group may have triggered stereotype threat in both experimental conditions, i.e., even when it was claimed in the control condition that the comparison had shown no group difference.

The results of a study by Hermann and Vollmeyer (2021) are in line with this interpretation. They found that girls' mathematics performance was impaired not only in an explicit stereotype threat condition in which boys were said to be better at mathematics than girls, but also in a condition in which participants were told that girls underperformed in mathematics only when confronted with the negative stereotype about their abilities. Only in a control condition in which the gender stereotype was not addressed (participants were told that only some individuals benefited from a positive attitude toward mathematics in their math performance) did girls perform equally well as boys. While one might have expected the stereotype threat to be lifted by making girls aware of the phenomenon, Hermann and Vollmeyer's (2021) findings suggest that the mere reference to the stereotype was sufficient to threaten girls. For the interpretation of our findings, this implies that explicitly stating that there was no difference in learning gains between students of German or other family language also triggered stereotype threat among students with Turkish family language due to implicit processes. This could explain why we found no difference in learning gains for students with Turkish family language between the stereotype activation and the control condition.

A different explanation why no difference in Turkish origin students' learning gains was observed between the two stereotype activation conditions is that in the control condition they may have wondered why the teacher explicitly mentioned that there was no difference in learning gains between the two language groups—as this implies that the Turkish group is usually assumed to be inferior. This interpretation is supported by our finding that across both experimental conditions students with Turkish family language perceived the teacher as more biased toward immigrant students than students from all other language backgrounds.

Yet another explanation why no stereotype threat effect was observed for students of Turkish descent is that they did not find the teacher's statement in the control condition credible according to which there was no difference in learning gains between language groups. As a result, students' learning gains may have been impaired to a similar extent in both the stereotype activation and the control condition. Support for this assumption is provided by our findings regarding students' perceptions of teacher bias. When the teacher said that there were no differences in learning gains between language groups, students with Turkish family language were more concerned that the teacher was biased than when she said that the groups differed. What is more, only when the teacher claimed there was no difference in learning gains were students with Turkish family language more concerned than students of other non-German language and more concerned than German origin students that the teacher was biased and not sensitive to immigrant students' needs.

#### Learning gains in immigrant students with non-Turkish family language

Building on the findings of Chaney et al. (2018), we had expected that the Turkish origin teacher, as a member of a similarly stigmatized outgroup, would also provide identity-safety to immigrant students with non-Turkish roots. Deviating from this hypothesis, students with other non-German family language suffered in their learning gains when the Turkish origin teacher voiced the stereotype but not when the German origin teacher did. In our stereotype activation condition, the psychological situation of students with Turkish family language differed from that of students with other non-German family language in two respects: The immigrant teacher belonged to the same (for students with Turkish family language) or a different minority group (for all other immigrant students), and the stereotype voiced by the teacher referred to the students' ingroup (for students with Turkish family language) or to immigrant students in general. While we can assume that students with Turkish family language are aware of the negative stereotype about people of Turkish descent in Germany (Asbrock, 2010; Froehlich & Schulte, 2019; Stang et al., 2021), the group of students with other non-German language in Germany is quite diverse and also includes immigrant groups to whom positive performance related stereotypes apply. For instance, Lorenz et al. (2016) observed that teachers in Germany hold positive achievement related stereotypes for students of Eastern European origin. Therefore, the teacher's statement in the stereotype activation condition that there was a difference between language groups was quite ambivalent for immigrant students with non-Turkish family language and more ambivalent than for students with Turkish family language, as there are both positive and negative associations with a non-German family language. It is possible that in this situation, immigrant students with a family language other than Turkish looked for a cue to resolve the ambiguity of the stereotype activation condition. Possibly, then, the Turkish origin teacher—as a member of a negatively stereotyped group—worked as a priming that students with non-German family language are inferior to those with German family language. Thus, stereotype activation by the Turkish origin teacher elicited identity-threat in immigrant students with non-Turkish family language resulting in lower learning gains. Thus, immigrant students with non-German family language suffering from stereotype threat only when instructed by the Turkish origin teacher could be due to students more likely interpreting the teacher's reference to a difference in learning gains between language groups as implying their own group was inferior when the teacher herself was a member of a negatively stigmatized group.

A different interpretation of the unexpected finding that non-Turkish immigrant students underperformed when instructed by the Turkish origin teacher is that they categorized the teacher as an ingroup member sharing their migration history only when she did not voice a potential identity-threat. When the teacher said that students' learning gains did not differ from those of German family language students, immigrant students with non-Turkish roots may have seen her as a member of a similarly stigmatized outgroup and thus as providing identity-safety (cf. Chaney et al., 2018), whereas when she said that there was a difference, she raised concerns that she might be biased against students of non-German language backgrounds and therefore students categorized her as an outgroup member. This interpretation is supported by our finding that immigrant students with non-German family language (but not students with Turkish family language) learned more vocabulary to the extent that they perceived the teacher as unbiased toward immigrant students' needs. If our interpretation is correct this would imply, however, that a minority ingroup expert model can prevent identity-threats only for stigmatized students of the same minority group—contradicting the findings by Chaney et al. (2018).

## Implications

What do the findings of our experimental studies entail regarding the public debate that increasing the proportion of teachers with an immigrant background could improve the situation for ethnic minority school students (e.g., Senatsverwaltung für Bildung, Jugend und Familie, 2020; Morgenroth et al., 2015; Syring et al., 2019)? Comparing the findings of our two studies we can say that the strong endorsement of the assumption that a minority expert role model is a “panacea for inequality” (Morgenroth et al., 2015, p. 465) we saw in our preservice teacher participants needs to be qualified, once the effects the minority teacher actually had on school students' psychological situation are taken into account. The pattern of findings for the two groups we studied was congruent in that both preservice teachers and school students perceived the Turkish teacher to be less demotivating than the German teacher when she said there were differences in learning outcomes between German language students and students with other family language (even though this pattern was only marginally significant in our school student sample). Our assumption was thus supported that in an identity-safe classroom a reference to differences in immigrant and non-immigrant students' learning prerequisites need not trigger stereotype threat. Only preservice teachers but not school students were more convinced of the Turkish minority than the German majority teacher's unbiasedness and student motivational support. Hence, the teacher being an ethnic minority member does not seem to be in itself an advantage for immigrant students, however, stigmatized students do profit from a minority teacher if there is an identity-threat.

A significant finding seems to us to be that students with Turkish family language overall were more skeptical than students of any other language group that the teacher would be biased toward immigrant students and that this difference disappeared when the teacher said that learning gains were different between students with Turkish versus German family language. It may be that the negative stereotype about Turkish people living in Germany (Asbrock, 2010; Froehlich & Schulte, 2019; Stang et al., 2021) is so pervasive and well known to students with Turkish roots that even a teacher from their ingroup does not automatically create identity-safety for them. Rather than the teacher's ethnicity, what mattered for the perception students with Turkish family language had of the teacher's unbiasedness was that she did not deny that the Turkish group is disadvantaged in their learning success.

While these findings do not provide direct support for the assumption that immigrant students profit from a minority teacher, increasing the percentage of teachers from various migration backgrounds is an important goal in itself as this would contribute to more diversity in the teacher staff and thus reflect the society's wish to include people of all ethnic and cultural groups. A more diverse teaching staff can also be expected to strengthen norms of cultural pluralism and multicultural beliefs among teachers of all backgrounds which in turn were found to be positively associated with ethnic minority and ethnic majority students' school belonging (Schachner, 2019), positive intergroup contact between ethnic minority and majority students (Schwarzenthal et al., 2018), and with less prejudice toward immigrant students (Hachfeld et al., 2012, 2015). The findings of the cited studies also suggest that in the long run a stronger representation of immigrant teachers will help eliminate negative stereotypes that prevail in German society against different groups of immigrants and thus reduce students' risk of becoming victims of stereotype threat at school.

## Limitations

Our research has several limitations. In our first study, only 50 preservice teachers had Turkish as their family language. While these participants were fairly evenly distributed across the four experimental conditions, cell sizes were of course quite small. These small sample sizes can possibly account for small and non-significant effect sizes in the MANOVA.

Furthermore, our second study was conducted during the COVID-19 pandemic, thus it was difficult to recruit school classes to participate. Consequently, we remained with rather small sample sizes which could have led to small effect sizes, and, in some cases, insufficient model fit of our regression models. Additionally, contact restrictions and school closings may have triggered distress in some of the students, which could have interacted with the identity-threat posed in some of our experimental conditions.

Furthermore, it might be surprising that we examined different perspectives namely the perception of preservice teachers and school students. Preservice teachers, on the one hand, were asked to anticipate school students' perception but might still have evaluated the teacher against professional standards, judging her behavior against that of an “ideal” teacher. School students, on the other hand, rated the effect of the teacher on themselves, possibly comparing her with the teachers in their own school. This could explain why school students' perception of the teacher but not the perception reported by preservice teachers differed depending on participants' own cultural background. Future research may want to investigate whether the perspectives would converge more if not only teachers but also students were asked how the teacher would affect students in general.

## Footnotes

1 The video tutorials were introduced to the literature by Ollrogge et al. (2022).

2 Our sample of school students included adolescents of Turkish ethnic minority students and adolescents of other non-German backgrounds. This begs the question whether the Turkish origin teacher is perceived as equally supportive by students with Turkish backgrounds and students with other non-German backgrounds and to what extent the Turkish origin teacher succeeds in counteracting stereotype threat effects among students of Turkish background and among students of other non-German background. We assume that the common experience of migration history is crucial and that it is less important whether teacher and student have the same ethnicity. Chaney et al. (2018) showed that an ingroup expert model buffers identity-threats for stigmatized learners even if the expert belongs to a different outgroup, as long as this outgroup is similarly stereotyped. In their studies, identity-threat effects for White women in STEM subjects were mitigated by a Black (rather than White) male expert, i.e., an expert from a similarly stereotyped outgroup, but not by an Asian male, i.e., an expert from an outgroup that was not similarly stereotyped. Glock und Schuchart (2020) argue that an immigrant student benefits from an immigrant teacher even if the teacher is not of their own ethnicity as they share the identity of not belonging to the ethnic majority. Supporting this view, Cortland et al. (2017) found that highlighting shared experiences of discrimination promoted positive relations between groups with shared ethnic minority identities. Accordingly, in our own research we expected that the Turkish origin teacher would buffer identity-threats emanating from the teacher referring to differences in learning gains between students with Turkish or German family language (stereotype activation) for immigrant students regardless of their respective ethnic backgrounds, as students feel identity-safe with the teacher either being a member of their own ethnic ingroup or a member of a similarly stereotyped ethnic minority group.

3 In the 2020/21 school year, 73.1% of general education teachers were female and 26.9% were male.

4 In order to protect the anonymity of the participants, we were only allowed to specify the five most common languages and one residual category as response options.

5 In all analyses, however, we used family language as reported by the students themselves at the end of the experiment as independent variable.

6 We report Cronbach's Alpha α for all measures incorporated as manifest variables but McDonalds Omega ω for all constructs included as latent constructs into our analyses. Moosbrugger and Kelava (2020) recommend using ω as a more strict alternative to α for reliability measurement in case of insufficient model fit in model-based methods of reliability estimation. For the competence measures in Study 2, no model-based reliability measure (ω) could be computed as answers were given dichotomously (correct/not correct).

7 We also computed the multiple group regression analysis including the subsample of students with German family language. Results indicated that, as expected, there were no effects of our experimental treatments on the posttest score for these students. Also, there were no significant changes in effect sizes in the other groups when including German language students.
